# Effects of vitamin D_3_ supplementation on oxidative stress and antioxidant enzyme after strenuous endurance exercise in healthy young men: a double-blind, placebo-controlled trial

**DOI:** 10.5114/biolsport.2025.139087

**Published:** 2024-07-31

**Authors:** Cheng-Tse Yang, Pei-Wei Weng, Li-Hui Chien, Sunil Kumar, Ming-Ta Yang

**Affiliations:** 1 Graduate Institute of Medical Sciences, Taipei Medical University, Taipei, Taiwan; 2 Department of Orthopaedics, School of Medicine, Taipei Medical University, Taipei, Taiwan; 3 International Ph.D. Program in Biomedical Engineering, Taipei Medical University, Taipei, Taiwan; 4 Graduate Institute of Athletics and Coaching Science, National Taiwan Sport University, Taoyuan, Taiwan; 5 Nottingham Law School (Patent), Nottingham Trent University, Nottingham, England; 6 Center for General Education, Taipei Medical University, Taipei, Taiwan; 7 Clinical Research Center, Taipei Medical University Hospital, Taipei, Taiwan

**Keywords:** Catalase, Glutathione peroxidase, Protein carbonylation, Superoxide dismutase, Thiobarbituric acid reactive substances

## Abstract

Vitamin D_3_ exhibits strong antioxidant properties. However, its effect on oxidative stress after strenuous endurance exercise remains unclear. Therefore, we investigated the effects of vitamin D_3_ supplementation on strenuous endurance-exercise-induced oxidative stress. In this study, 26 male participants consumed vitamin D_3_ (5,000 IUs of vitamin D_3_ daily for 4 weeks) or placebo. After four weeks, all participants performed strenuous endurance exercise at 65% of $\dot{\text{V}}$O_2max_. Blood samples were collected before and after four weeks of supplementation to determine the participants’ serum 25(OH)D concentration. Additionally, venous blood samples were collected before, immediately after, two hours after, and 24 hours after the strenuous endurance exercise test to analyze markers of oxidative damage (thiobarbituric acid reactive substances, and protein carbonylation) and antioxidant enzyme activity (catalase, glutathione peroxidase, and superoxide dismutase). After four weeks of vitamin D_3_ supplementation, the concentrations of protein carbonylation and superoxide dismutase at post-0-h, post-2-h, and post-24-h were significantly higher in the placebo group than in the vitamin D_3_ group (all p < 0.05); the concentration of thiobarbituric acid reactive substances was significantly higher in the placebo group than in the vitamin D_3_ group at post-24-h (p < 0.05); and the concentration of catalase was significantly higher in the placebo group than in the vitamin D_3_ group at post-0-h (p < 0.05). Our results indicated that four weeks of 5,000-IU vitamin D_3_ supplementation (oil form) effectively increased the participants’ serum 25(OH)D concentration and mitigated the accumulation of strenuous-endurance-exercise-induced markers of oxidative stress (e.g., thiobarbituric acid reactive substances and protein carbonylation).

## INTRODUCTION

Reactive oxygen species (ROS) encompasses various oxygen-containing free radicals, including superoxide anion (${\text{O}}_{2}^{•-}$), hydroxyl radical (•OH), and hydrogen peroxide (H_2_O_2_) [1]. ROS is derived from endogenous sources, such as nicotinamide adenine dinucleotide phosphate (NADPH) oxidase on plasma membranes, myeloperoxidase in phagocytes, and mitochondrial respiratory chain function; they can also originate from exogenous sources, such as ageing, inflammation, radiation, and toxic chemicals [2]. Because of their high reactivity and instability, excessive amounts of ROS may cause deleterious oxidative damage to membrane lipids (measured by assessing the concentration of thiobarbituric acid reactive substances [TBARS]) and proteins (measured by assessing protein carbonylation [PC]), consequently leading to cell death [3]. Furthermore, ROS participates in the pathology of numerous diseases, such as cancer, atherosclerosis, Alzheimer’s disease, and pulmonary diseases [1]. The human body counteracts free radicals through enzymatic antioxidant systems that function to prevent the formation of free radicals, including glutathione peroxidase (GP_X_), superoxide dismutase (SOD), and catalase (CAT) [2]. Given the afore-mentioned findings, maintaining the oxidant/antioxidant equilibrium is essential for preventing various adverse effects [4]. Accordingly, the concept of maintaining an oxidant/antioxidant equilibrium has been widely applied and recognized as oxidative stress [5], and numerous studies have explored the elimination of oxidative damage [6].

Living an active lifestyle that includes moderate exercise has been demonstrated to prevent various diseases and improve antioxidant defenses [7]. Under normal exercise training, ROS levels do not reach detrimental levels due to exercise-induced fatigue. However, during strenuous exercise training, ROS can reach a detrimental level, a phenomenon proposed as hormesis [5]. Intense exercise produces high levels of ROS that cannot be entirely counteracted by the antioxidant system, and the high levels of ROS may impair cellular function and even limit exercise performance [8]. During intense exercise, several major factors contribute to the production of high levels of ROS, including ischemia/reperfusion, increased mitochondrial activity, and the activation of enzymes in the muscles [5]. A study reported that exercising at an intensity exceeding 50% of $\dot{\text{V}}$O_2max_ increases ROS concentrations beyond the typical physiological range; it also indicated that the concentrations of these compounds are influenced by various factors, including age, gender, diet, and training status [9]. Additionally, a study demonstrated that running for 90 min at 65% of $\dot{\text{V}}$O_2max_ followed by a 15-min eccentric training phase led to increases in the concentrations of serum lipid peroxides, PC, and 8-iso-prostane [10]. Therefore, the exercise protocol implemented in our study comprised two hours of cycling performed at 65% of $\dot{\text{V}}$O_2max_ and 70 rpm (as measured using a cycle ergometer).

Vitamin D, an essential vitamin, exists in two forms, namely vitamin D_2_ and vitamin D_3_. Vitamin D_2_ is mostly present in plants, yeast, and fungi, whereas vitamin D_3_ is found in animal-based foods such as salmon, tuna, sardine, and liver [11]. Alternatively, the human body can produce vitamin D upon exposure to ultraviolet light (UV) in the band B range, which is why vitamin D is commonly referred to as the sunshine vitamin [12]. The active form of vitamin D, 1,25(OH)_2_D, regulates a series of physiological functions, including the immune system, bone health, cardiac function, muscle damage, and oxidative stress [6, 13, 14].

Vitamin D_3_ was first identified as a potential antioxidant by Wise-man in 1993, who suggested that the hydrophobic portions of 1,25(OH)_2_D_3_ can reduce membrane viscosity, thereby protecting the cell membrane from free radicals and lipid peroxidation [15]. In addition, vitamin D deficiency has been reported to cause mitochondrial dysfunction, leading to ROS production [16]. Studies have also demonstrated that vitamin D deficiency promotes lipid and protein oxidation in the skeletal muscle and alters antioxidant enzyme activity [17, 18]. Ke et al. randomized rats into four groups: a control group, a vitamin D_3_ supplementation group, an exercise intervention group, and a group subjected to both vitamin D_3_ supplementation and exercise intervention. After the rats in the exercise intervention groups completed a high-intensity exercise intervention, all rats were injected with normal saline or vitamin D_3_ (1 ng/mL). Compared with the rats in the exercise-only group, those in the group subjected to vitamin D_3_ supplementation combined with the exercise intervention exhibited significantly decreased concentrations of the oxidative product 4-hydroxynonenal in their kidneys and lungs [19]. In another study, healthy men received 3,600 IUs of daily vitamin D_3_ supplementation for two weeks; thereafter, their oxidative stress and antioxidant indices during elastic-band resistance training were measured; the results indicated that vitamin D_3_ supplementation did not significantly alter oxidative stress markers and antioxidant enzyme concentrations [20].

Studies examining the effects of vitamin D_3_ supplementation on oxidative stress have reported inconsistent results, with some indicating a reduction in oxidative stress and others failing to identify any significant effects. In addition, the clinical translation of these in vitro and in vivo results remains unclear. To address these questions, we conducted a trial to evaluate the effects of 5,000 IUs of daily vitamin D_3_ supplementation over four weeks on strenuous endurance exercise (SEE)-induced oxidative stress.

## MATERIALS AND METHODS

### Participants

The statistical power analysis software program, G*Power (v. 3.1.9.7), was used to calculate the sample size in the present study. With a 2 × 4 two-way mixed-design analysis of variance (ANOVA), an effect size of 0.4, an α value of 0.05, and a power of 0.95, the appropriate sample size was determined to be 16. Therefore, our study recruited 26 healthy men without any underlying diseases (e.g., cardiovascular disease, diabetes, kidney disease, liver disease, and autoimmune disease). Sex differences are apparent in oxidative stress response and vitamin D concentrations. Therefore, we opted to include only male participants to minimize variation in the study [21, 22]. Young Taiwanese males aged between 20 and 40 were invited to participate in the study through word-of-mouth invitations at Taipei Medical University. Individuals with acute sports injuries, a blood 25(OH)D concentration of > 30 ng/mL, and a $\dot{\text{V}}$O_2max_ of < 40 mL/kg/min were excluded. Individuals who participated in other research programs or experiments within the three months preceding the start of the present study were also excluded. The purpose and process of the present experiment were thoroughly explained to eligible individuals, after which they were asked to complete a health status questionnaire and sign an informed consent form. During the experimental period, all participants were instructed to refrain from alcohol consumption, maintain a normal lifestyle, and avoid taking other dietary supplements. Our study was conducted at Taipei Medical University between March and May 2021 to minimize the variability associated with UV exposure. The present study was approved by the Joint Institutional Review Board of Taipei Medical University (IRB number: N202010026) on November 5, 2020, and its procedures were conducted as the principles in the Declaration of Helsinki. Our clinical trial was also registered at Clinical Trial.gov (ID number: NCT05779410). Table 1 provides information on the attributes of the participants.

**TABLE 1:** Participant characteristics

Variable	Vitamin D_3_ Group	Placebo Group
Age (years)	21.0 (2.5)	22.0 (4.0)
Height (cm)	174.0 (12.5)	175 (11.0)
Body weight (kg)	63.0 (15.0)	70.0 (12.0)
Body mass index (kg/m^2^)	22.0 (3.8)	22.8 (2.6)

Data are presented as median (interquartile range, IQR); n = 13 in each group.

### Experimental design

**FIG. 1:**
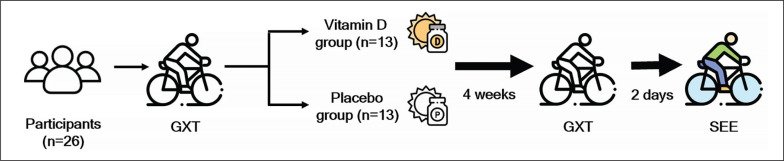
Experimental scheme. Abbreviations: GXT, graded exercise test; SEE, strenuous endurance exercise.

For the present study, a double-blinded matched-pair design was adopted, and 26 participants were divided into a placebo (n = 13) group and a vitamin D_3_ (n = 13) group on the basis of their $\dot{\text{V}}$O_2max_ levels. The participants were required to complete a graded exercise test (GXT) on a cycle ergometer until exhaustion. After 4 weeks of vitamin D_3_ or placebo supplementation, all participants completed a second GXT until exhaustion, and with the collected $\dot{\text{V}}$O_2max_ data as a reference of the participants’ exercise intensity (65% of $\dot{\text{V}}$O_2max_) when they performed SEE 2 days after the second GXT. All participants were required to fast and consume a standardized meal (639 kcal, 105.4 g carbohydrates, 16.4 g fat, 17.6 g protein) 1.5 hours before exercise. Venous blood was drawn before, immediately after, 2 h after, and 24 h after the SEE test to identify changes in the concentrations of markers of oxidative damage (TBARS and PC) and the activity of antioxidant enzymes (GPx, SOD, and CAT). The experiment flowchart is illustrated in Figure 1.

### Supplementation

While a daily dose of 4,000 IU of vitamin D is considered the tolerable upper intake level for adults in some authorities, the Endocrine Society recommends treating adults with vitamin D deficiency (< 30 ng/mL) with 50,000 IU of vitamin D_2_ or D_3_ once a week for eight weeks to achieve a blood level of 25(OH)D above 30 ng/mL [23]. Besides, the dose of 5,000 IU of vitamin D is half the upper limit of the recommended intake for adults (19–70 years) according to the Endocrine Society. Additionally, research suggests that the body utilizes an average of 3,000–5,000 IU of vitamin D daily [24]. Furthermore, the population we are specifically targeting vitamin D-deficient athletes who participate in strenuous endurance exercise, which may warrant different supplementation recommendations. Therefore, the supplementation dosage and method used in this study were consistent with those employed in another study that successfully raised vitamin D levels from insufficient to sufficient while considering safety [25]. In the present experiment, the individuals in the supplementation group consumed 5,000 IUs of liquid-form vitamin D_3_ (Liquid Shield Vitamin D_3_+E, Panion & BF Biotech, Taipei, Taiwan) daily after lunch for four weeks. By contrast, the placebo group consumed medium-chain triglycerides with the same color, taste, and odor as the vitamin D_3_ supplement (Panion & BF Biotech). Through regular messages, all participants were reminded to take the supplement capsules as prescribed and were monitored to ensure that the vitamin D supplementation did not lead to any adverse effects. Compliance was assessed by calculating the number of leftover capsules.

### Blood sampling and analysis

Before and after the vitamin D_3_ intervention, 1 mL of blood sample was drawn from the antecubital vein of each participant and centrifuged at 3,000 rpm for 10 min at 4°C (Centrifuge 5702 R; Eppendorf; Hamburg, Germany). To measure the 25(OH)D concentration, the serum obtained after centrifugation was collected and tested on an automated immunoassay analyzer (Cobas e801; Roche Diagnostics, Mannheim, Germany) at the Taipei Medical University Hospital.

From the participants, additional blood samples (1 mL) were drawn from the antecubital vein and collected in ethylenediaminetetraacetic acid tubes before (pre-SEE), immediately after (post-0), two h after (post-2), and 24 h after (post-24) the SEE test. The ethylenediami-netetraacetic acid tubes were centrifuged at 3,000 rpm for 10 min at 4°C (Eppendorf Centrifuge 5702 R), and the supernatant was collected in microcentrifuge tubes, stored at −80°C, and subsequently analyzed for TBARS, PC, GP_X_, SOD, and CAT concentrations.

The TBARS concentration was measured using colorimetric methods. Specifically, 200 μL of plasma or the 1,1,3,3-tetramethoxypropane standard was mixed with 1 mL of the TBARS reagent (15% trichloroacetic acid and 0.38% thiobarbituric acid in 0.25 N HCl). The tubes were then placed in a dry bath set to a temperature of 100°C for 30 min. Thereafter, the tubes were cooled on ice for 10 min and centrifuged (3,900 rpm) at room temperature for 10 min. Next, 200 μL of the supernatant was transferred to microplate wells, and absorbance was read at 535 nm. PC were quantified using a commercial enzyme-linked immunoassay protein carbonylation kit (Cat. no ab238536; Abcam, Cambridge, MA, USA) as per the manufacturer’s instructions. SOD analysis was performed using a SOD determination kit (Cat. no 19160; Sigma-Aldrich, St. Louis, MO, USA) as per the manufacturer’s instructions. In addition, CAT and GPx concentrations were also measured using an assay kit (Cat. no 707002; Cat. no 703102; Cayman, Ann Arbor, MI, USA) as per the manufacturer’s instructions.

### Graded exercise test (GXT) protocol

The participants completed a GXT to establish a reference for determining the intensity of the SEE test (65% $\dot{\text{V}}$O_2max_). The GXT was conducted on a cycle ergometer (Monark LC6, Monark Exercise AB, Sweden). After a 5-min warm-up (50 W, 70 ± 5 rpm), the workload was increased by 25 W every 2 min. The investigators provided strong verbal encouragement and instructed the participants to maintain a 70-rpm pedaling rate until they could no longer maintain a 60-rpm pedaling rate. The participants’ carbon dioxide elimination ($\dot{\text{V}}$O_2_), oxygen consumption ($\dot{\text{V}}$O_2_), and exchange volume were measured using a mobile gas analyzer (MetaMax 3B; Cortex Biophysik, Leipzig, Germany), and their heart rates were measured using a heart rate monitor (Polar S610, Kempele, Finland). In each stage, the rating of perceived exertion of the participants was measured using a standard 6–20 Borg scale. Volitional exhaustion was defined when at least two of the three criteria were fulfilled as follows: (1) the maximum heart rate is maintained within 15 beats/min of the predicted value, (2) the respiratory exchange ratio is > 1.1, and (3) rating of perceived exertion is ≥ 18.

### Strenuous endurance exercise (SEE) test

The participants underwent the SEE test 2 days after completing the GXT test. After arriving at the laboratory, and rested for 30 min and wore a heart rate monitor (S610; Polar, Kempele, Finland) for subsequent heart rate monitoring during the SEE test. After performing a 5-min warm-up routine (heart rate ≤ 150 beats/min), each participant cycled on a cycle ergometer at 65% of $\dot{\text{V}}$O_2max_ and 70 rpm for 2 h. The $\dot{\text{V}}$O_2_ of the participants was monitored during the GXT test. If a participant failed to maintain oxygen consumption at 65% of $\dot{\text{V}}$O_2max_, the workload of the cycle ergometer was adjusted. During the exercise test, water was provided ad libitum (< 300 mL) every 15 min. At the end of the 2-h cycling test, each participant was requested to increase their pedaling rate to 100 rpm until they could no longer maintain a 90-rpm pedaling rate (i.e., exhaustion).

### Statistical analysis

The normality of data distribution was evaluated using the Shapiro–Wilk test. As some parameters were not normally distributed, non-parametric methods were employed, and median and interquartile range (IQR) were used to present all data. Participant characteristics and serum 25(OH)D concentrations were analyzed using the Mann–Whitney U test and Wilcoxon signed-rank test. The significance of differences between the vitamin D_3_ group and the placebo group for other variables was analyzed using the Mann–Whitney U test. Different time points were statistically compared using the Friedman test and Wilcoxon signed-rank test as a post hoc test. Statistical significance was set at p < 0.05. Cohen’s d values were used to estimate effect sizes, which were categorized as small (0.20–0.49), moderate (0.50–0.79), and large (≥ 0.80). When p < 0.05, the data sets were regarded as statistically significant. All statistical analyses were performed using SPSS version 22.0 (IBM, Armonk, NY, USA).

## RESULTS

### Effects of vitamin D_3_ supplementation on serum 25(OH)D concentration

**FIG. 2:**
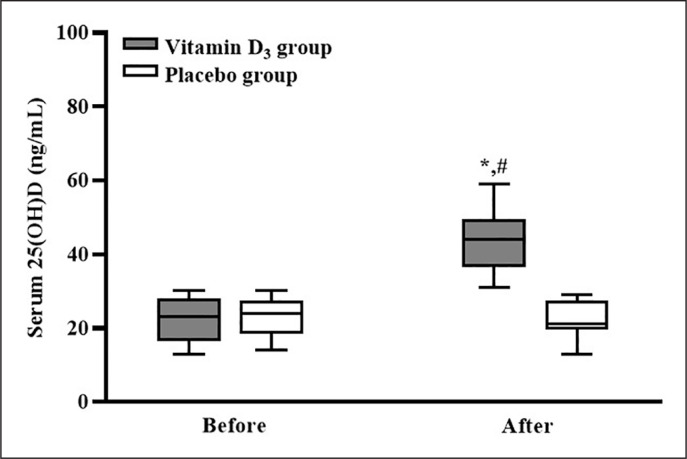
Serum 25(OH)D concentration before and after 4-week supplementation. * p < 0.05 compared with concentration before supplementation. # p < 0.05 compared with serum 25(OH)D concentration in placebo group.

After counting the leftover capsules, the adherence to supplementation was 100%. The serum 25(OH)D concentrations of the participants in the placebo and vitamin D_3_ groups before and after vitamin D_3_ supplementation are listed in Figure 2. In the placebo group, the participants’ serum 25(OH)D concentrations before and after supplementation were not significantly different (p > 0.05). In the vitamin D_3_ group, the participants’ serum 25(OH)D concentration was significantly higher after supplementation than before supplementation (p < 0.05), and the serum 25(OH)D concentration of the vitamin D_3_ group was significantly higher than that of the placebo group (p < 0.05; *d* = 3.12). Our results indicated that four weeks of vitamin D_3_ supplementation effectively increased the serum 25(OH) D concentration.

### Effects of vitamin D_3_ supplementation on oxidative damage

**FIG. 3:**
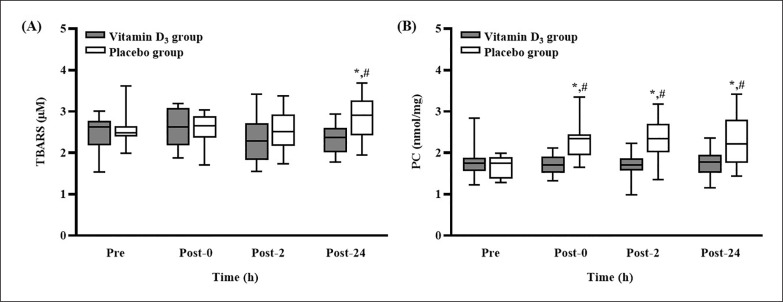
Changes in (A) TBARS and (B) PC concentrations after strenuous endurance exercise. * p < 0.05 compared with baseline concentration. # p < 0.05 compared with concentration in vitamin D_3_ group. Abbreviations: TBARS, thiobarbituric acid reactive substances; PC, protein carbonylation; Pre-SEE, before exercise; Post-0, immediately after exercise; Post-2, 2 h after exercise; Post-24, 24 h after exercise.

The TBARS and PC concentrations after 4 weeks of vitamin D_3_ or placebo supplementation are presented in Figure 3. The TBARS concentration in the placebo group was significantly higher at post-24 than at pre-SEE (p < 0.05); at post-24, it was significantly higher than that in the vitamin D_3_ group (p < 0.05, *d* = 1.03) (Figure 3A). In addition, the concentration of PC in the placebo group was significantly higher at post-0, post-2, and post-24 than at pre-SEE (all p < 0.05); it was also significantly higher than that in the vitamin D_3_ group at the three post-SEE time points (all p < 0.05; *d* = 1.06 [post-0], 1.00 [post-2], and 0.91 [post-24]) (Figure 3B).

### Effects of vitamin D_3_ supplementation on antioxidant enzyme concentrations

**FIG. 4:**
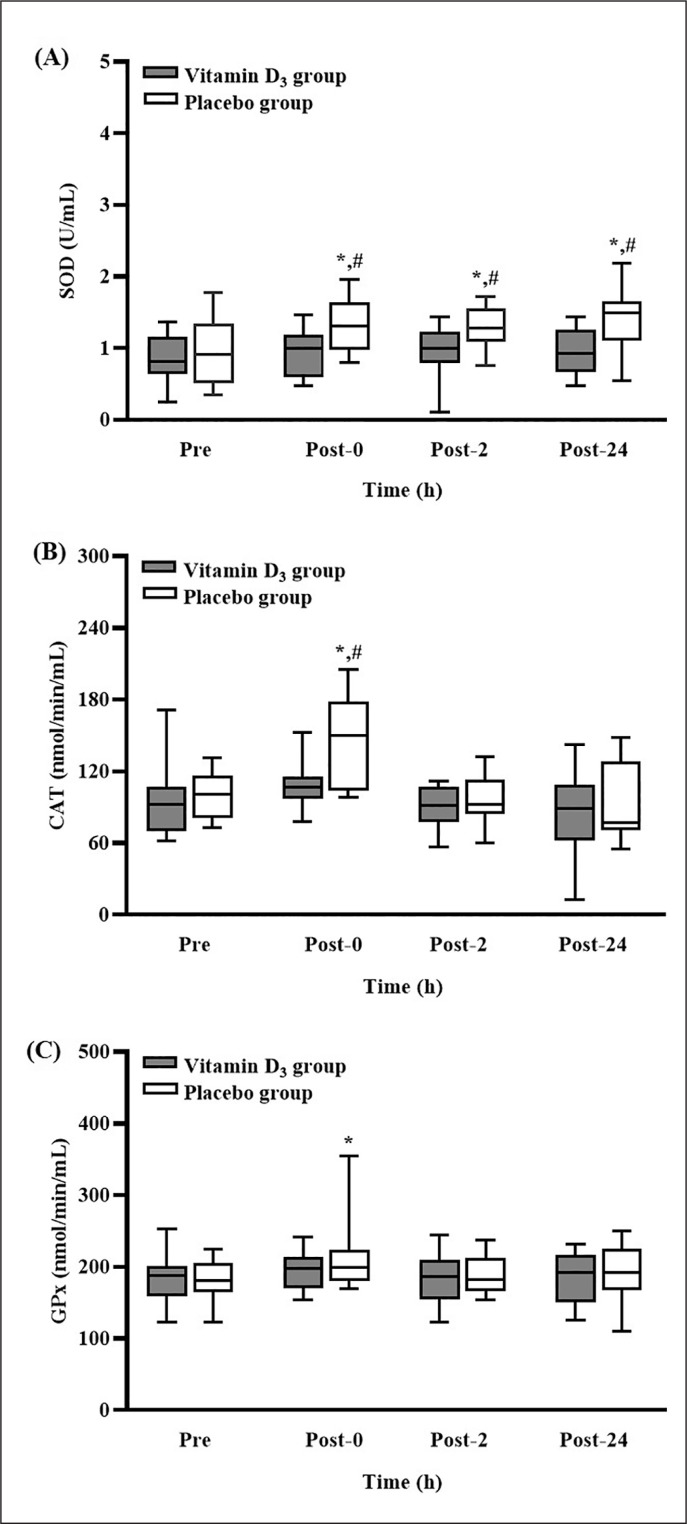
Changes in (A) SOD, (B) CAT, and (C) GPx concentrations after strenuous endurance exercise. * p < 0.05 compared with baseline concentration. # p < 0.05 compared with concentration in vitamin D_3_ group. Abbreviations: SOD, superoxide dismutase; CAT, catalase; GPx, glutathione peroxidase; Pre-SEE, before exercise; Post-0, immediately after exercise; Post-2, 2 h after exercise; Post-24, 24 h after exercise.

The levels of SOD, CAT, and GPx activity after 4-week vitamin D_3_ or placebo supplementation are presented in Figure 4. In the placebo group, the level of SOD activity was significantly higher at post-0, post-2, and post-24 than at pre-SEE (all p < 0.05). Moreover, the level of SOD activity in the placebo group was significantly higher than that in the vitamin D_3_ group at the three post-SEE time points (all p < 0.05; *d* = 1.13 [post-0], 1.00 [post-2], and 1.19 [post-24]) (Figure 4A). In the placebo group, the level of CAT activity was significantly higher at post-0 than at pre-SEE (p < 0.05). Moreover, the level of CAT activity in the placebo group was significantly higher than that in the vitamin D_3_ group at post-0 (p < 0.05; *d* = 0.94) (Figure 4B). Nevertheless, the level of GPx activity in the placebo group was significantly higher at post-0 than at pre-SEE (p < 0.05) (Figure 4C).

## DISCUSSION

According to the US Endocrine Society, vitamin D insufficiency is defined as a 25(OH)D of 21–29 ng/ml [23]. After four weeks of 5,000-IU daily vitamin D_3_ supplementation, the serum 25(OH)D concentration of the participants in the vitamin D_3_ group significantly increased from an insufficient level (median: 23 ng/mL) to a sufficient level (median: 44 ng/mL). This result is consistent with other studies, which reported that four weeks of 5,000-IU daily vitamin D_3_ supplementation effectively increased serum 25(OH)D concentrations to mitigate vitamin D deficiency [14, 25]. Thus, four weeks of 5,000-IU daily vitamin D_3_ supplementation is an appropriate strategy for increasing the serum 25(OH)D concentration in vitamin D insufficient (< 30 ng/mL) athletes.

In the placebo group, the concentration of TBARS was significantly elevated at post-24, and the concentration of PC was significantly elevated at post-0, post-2, and post-24. These results indicate that the SEE protocol induced significant oxidative damage. Studies have reported that SEE significantly increased the concentrations of TBARS and PC [10, 13]. However, in the present study, the concentration of TBARS was not significantly elevated at post-0 and post-2. In a study that examined exercise-induced muscle damage, the blood concentration of TBARS peaked at post-24 in the placebo group [26]. Additionally, research has demonstrated that aerobic exercise increases the likelihood of protein oxidation and carbonylation formation. By contrast, rigorous isometric exercise increases the likelihood of lipid peroxidation [27]. These findings may explain why only the concentration of PC and not that of TBARS was significantly elevated at post-0 and post-2 in the present study. Furthermore, our laboratory indicated that our intense exercise protocol induced muscle damage [14]. PC have also been demonstrated to exhibit the highest correlation coefficients with muscle damage, and this association also contributes to the discrepancy [28]. Additionally, in the present study, the concentration of PC in the placebo group was significantly higher than that in the vitamin D_3_ group at post-0, post-2, and post-24. The concentration of TBARS in the placebo group was significantly higher than that in the vitamin D_3_ group at post-24, suggesting that vitamin D_3_ supplementation attenuated oxidative damage. Studies have demonstrated that an increased blood 25(OH)D concentration can significantly reduce the levels of various markers of oxidative damage under specific conditions (e.g., vitamin D–deficient diet, low back pain, and polycystic ovary syndrome) [17, 18, 29]. Furthermore, given that excessive levels of ROS cause secondary muscle damage [30], our results correspond to those of another study, which suggested that vitamin D_3_ supplementation reduces ROS production, leading to reduced SEE-induced muscle damage [14]. The present study is the first human study to report that vitamin D_3_ supplementation can significantly prevent SEE-induced oxidative damage. Therefore, four weeks of 5,000-IU daily vitamin D_3_ supplementation is an appropriate strategy for alleviating SEE-induced oxidative damage in vitamin D insufficient (< 30 ng/mL) athletes.

In the present study, the level of SOD activity in the placebo group was significantly higher at post-0, post-2, and post-24 than at pre-SEE. In the same group, the levels of CAT and GPx activity were significantly higher at post-0 than at pre-SEE. The results for the markers of oxidative damage indicated a significant increase in the antioxidant system activity in the placebo group with oxidative damage but not in the vitamin D_3_ group without oxidative damage. In the present study, antioxidant enzymes were activated only when oxidative damage occurred, which aligns with the findings of another study [31]. Similarly, a study reported that intense exercise increased the activity of antioxidant enzymes, which counteracted oxidative stress [32]. Alternatively, a study suggested that vitamin D_3_ exhibits antioxidant properties that can mitigate oxidative damage. These properties can be attributed to several mechanisms. First, a previous study demonstrated that vitamin D contains hydrophobic parts that can stabilize and protect the membrane from lipid peroxidation [15]. Additionally, an in vitro study conducted in 2005 suggested that vitamin D_3_ exerts a stronger antioxidant effect on zinc-induced oxidative stress than vitamin E, β-estradiol, and melatonin [33]. Second, researchers have argued that vitamin D reduces the gene expression of NADPH oxidase, which is the primary generator of ROS [34]. Finally, a study indicated that vitamin D mediates ROS production through the Nrf2-ARE-signaling pathway by increasing the activity of nuclear factor-erythroid factor 2-related factor 2, which is a transcription factor that controls the basal and induced expression of a series of antioxidant response elements [35]. Therefore, our results suggest that vitamin D_3_ supplementation is adequate for managing SEE-induced oxidative stress, possibly because of its inherent antioxidant capacity. However, the exact mechanism by which vitamin D_3_ mediates oxidative stress is still unclear. Additional research is required to clarify the molecular mechanisms underlying the effects of vitamin D_3_ supplementation on SEE-induced oxidative stress.

At present, the in vivo findings regarding the antioxidant effects of vitamin D on skeletal muscle are limited. In a study by Dzik et al., patients with both low back pain and vitamin D deficiency consumed 3,200 IUs of vitamin D for five weeks, and they experienced significant decreases in the concentrations of 8-isoprostanes and PC in their paraspinal muscle along with significant decreases in cytosolic SOD and GPx activity; these finding [18] are similar to those of our study. However, in other studies examining either rats or human participants with other diseases, the opposite effect was reported, suggesting that vitamin D_3_ supplementation increases antioxidant activity [17, 36]. The differences between the findings of these studies may be due to species differences (humans vs. rats), muscle pathology, or differences in the sources of oxidative damage. As was discussed in an earlier part of the present study, we assumed that vitamin D deficiency increased oxidative stress, thereby leading to increased SOD, CAT, and GPx activity; furthermore, vitamin D_3_ supplementation improved oxidative damage and the balance of the antioxidant system. Nevertheless, human studies should be conducted to further clarify the effects of vitamin D on the antioxidant status and the related mechanisms.

Maintaining an adequate concentration of serum 25(OH)D is essential for athletes. A study recommended a blood 25(OH)D concentration of > 40 ng/mL for athletes because this is the threshold for the onset of vitamin D storage in muscles and fat [24]. Under such circumstances, liver hydroxylation shifts to zero-order kinetics, and tissue calcitriol levels depend on skin synthesis or oral intake below this range, indicating substrate starvation [37]. Similarly, we demonstrated that serum 25(OH)D concentrations of ≥ 42 ng/mL had a positive effect on SEE-induced oxidative damage immediately after exercise. However, our previous study revealed that serum 25(OH)D concentrations of ≥ 44 ng/mL did not affect the time to exhaustion and $\dot{\text{V}}$O_2max_ even under vitamin D_3_ supplementation [14]. In addition, a study indicated that achieving serum 25(OH)D concentrations of > 48 ng/mL is necessary for achieving extra-skeletal health benefits [38]. Therefore, the optimal serum 25(OH)D concentration that athletes should achieve through vitamin D_3_ supplementation remains unclear. Future studies should develop an optimal vitamin D_3_ supplementation strategy for athletes.

The strength of this study lies in its status as the first human study to explore the effects of relatively higher vitamin D_3_ supplementation doses on oxidative stress and antioxidant enzymes, demonstrating that vitamin D supplementation can prevent exercise-induced oxidative damage. However, the study has limitations, including a lack of assessment for indicators of adverse effects such as hypercalcemia. Previous studies have shown that calcium levels remain normal with a daily dose of 8,000 IU after 12 weeks of supplementation, with similar participant characteristics [39]. Additionally, the study did not include fat mass data in the participant characteristics and had a limitation of lower BMI, which may have minor effects on vitamin D metabolism. Nevertheless, there were no significant BMI differences between the two groups, suggesting similar anthropometric conditions to some extent.

## CONCLUSIONS

Our results indicate that 5,000 IUs of oral vitamin D_3_ supplementation (oil form) for 4 weeks effectively increases the serum 25(OH)D concentration to prevent oxidative damage (TBARS and PC). Our study preliminarily supports the implementation of a nutritional supplementation strategy that can mitigate SEE-induced oxidative damage in athletes performing intensive training. Therefore, future studies should investigate the potential cellular mechanism by which vitamin D_3_ reduces SEE-induced oxidative damage as well as the effects of vitamin D_3_ supplementation at various dosages or over various periods.

## Data Availability

The data sets generated in this study are available from the corresponding author on reasonable request.

## References

[1] Forman HJ, Zhang H. Targeting oxidative stress in disease: promise and limitations of antioxidant therapy. Nat Rev Drug Discov. 2021; 20(9):689–709. 34194012 10.1038/s41573-021-00233-1 PMC8243062

[2] Morry J, Ngamcherdtrakul W, Yantasee W. Oxidative stress in cancer and fibrosis: opportunity for therapeutic intervention with antioxidant compounds, enzymes, and nanoparticles. Redox Biol. 2017; 11:240–53. 28012439 10.1016/j.redox.2016.12.011 PMC5198743

[3] Kowalczyk P, Sulejczak D, Kleczkowska P, Bukowska-Ośko I, Kucia M, Popiel M, et al. Mitochondrial oxidative stress&mdash; a causative factor and therapeutic target in many diseases. Int J Mol Sci. 2021; 22(24):13384. 34948180 10.3390/ijms222413384 PMC8707347

[4] Galli D, Carubbi C, Masselli E, Vaccarezza M, Presta V, Pozzi G, et al. Physical activity and redox balance in the elderly: signal transduction mechanisms. Appl Sci. 2021; 11(5):2228.

[5] Powers SK, Deminice R, Ozdemir M, Yoshihara T, Bomkamp MP, Hyatt H. Exercise-induced oxidative stress: friend or foe? J Sport Health Sci. 2020; 9(5):415–425. 32380253 10.1016/j.jshs.2020.04.001 PMC7498668

[6] Kawamura T, Muraoka I. Exercise-induced oxidative stress and the effects of antioxidant intake from a physiological viewpoint. Antioxidants. 2018; 7(9):119. 30189660 10.3390/antiox7090119 PMC6162669

[7] Simioni C, Zauli G, Martelli AM, Vitale M, Sacchetti G, Gonelli A, et al. Oxidative stress: Role of physical exercise and antioxidant nutraceuticals in adulthood and aging. Oncotarget. 2018; 9(24):17181–17198. 29682215 10.18632/oncotarget.24729 PMC5908316

[8] D’Angelo S. Polyphenols: potential beneficial effects of these phytochemicals in athletes. Curr Sport Med Rep. 2020; 19(7):260–265. 10.1249/JSR.0000000000000729 32692061

[9] Gancitano G, Reiter RJ. The multiple functions of melatonin: applications in the military setting. Biomedicines. 2022; 11(1):5. 36672513 10.3390/biomedicines11010005 PMC9855431

[10] Morawin B, Turowski D, Naczk M, Siatkowski I, Zembron-Lacny A. The combination of α-lipoic acid intake with eccentric exercise modulates erythropoietin release. Biol Sport. 2014; 31(3):179–85. 25177095 10.5604/20831862.1111435 PMC4135061

[11] Aji AS, Yerizel E, Desmawati, Lipoeto NI. The association between lifestyle and maternal vitamin D during pregnancy in West Sumatra, Indonesia. Asia Pac J Clin Nutr. 2018; 27(6):1286–1293. 30485928 10.6133/apjcn.201811_27(6).0016

[12] Small AG, Harvey S, Kaur J, Putty T, Quach A, Munawara U, et al. Vitamin D upregulates the macrophage complement receptor immunoglobulin in innate immunity to microbial pathogens. Commun Biol. 2021; 4(1):401. 10.1038/s42003-021-01943-3 PMC7994403 33767430

[13] Calella P, Cerullo G, Di Dio M, Liguori F, Di Onofrio V, Gallè F, et al. Antioxidant, anti-inflammatory and immunomodulatory effects of spirulina in exercise and sport: a systematic review. Front Nutr. 2022; 9:1048258. 36590230 10.3389/fnut.2022.1048258 PMC9795056

[14] Liu M-C, Weng P-W, Chen S-C, Liu T-H, Huang H-W, Huang C-T, et al. Immunologic, anti-inflammatory, and anti-muscle damage profile of supplemented vitamin D_3_ in healthy adults on strenuous endurance exercise. Biology. 2023; 12(5):657. 37237471 10.3390/biology12050657 PMC10215688

[15] Wiseman H. Vitamin D is a membrane antioxidant. Ability to inhibit iron-dependent lipid peroxidation in liposomes compared to cholesterol, ergosterol and tamoxifen and relevance to anticancer action. FEBS Lett. 1993; 326:285–288. 8325381 10.1016/0014-5793(93)81809-e

[16] Latham CM, Brightwell CR, Keeble AR, Munson BD, Thomas NT, Zagzoog AM, et al. Vitamin D promotes skeletal muscle regeneration and mitochondrial health. Front Physiol. 2021; 12:660498. 33935807 10.3389/fphys.2021.660498 PMC8079814

[17] Bhat M, Ismail A. Vitamin D treatment protects against and reverses oxidative stress induced muscle proteolysis. J Steroid Biochem Mol Biol. 2015; 152:171–179. 26047554 10.1016/j.jsbmb.2015.05.012

[18] Dzik K, Skrobot W, Flis DJ, Karnia M, Libionka W, Kloc W, et al. Vitamin D supplementation attenuates oxidative stress in paraspinal skeletal muscles in patients with low back pain. Eur J Appl Physiol. 2018; 118(1):143–151. 29143122 10.1007/s00421-017-3755-1

[19] Ke CY, Yang FL, Wu WT, Chung CH, Lee RP, Yang WT, et al. Vitamin D_3_ reduces tissue damage and oxidative stress caused by exhaustive exercise. Int J Med Sci. 2016; 13(2):147–153. 26941574 10.7150/ijms.13746 PMC4764782

[20] Kalvandi F, Azarbayjani MA, Azizbeigi R, Azizbeigi K. Elastic resistance training is more effective than vitamin D_3_ supplementation in reducing oxidative stress and strengthen antioxidant enzymes in healthy men. Eur J Clin Nut. 2022; 76(4):610–615. 10.1038/s41430-021-01000-6 PMC8431951 34508257

[21] Tower J, Pomatto LCD, Davies KJA. Sex differences in the response to oxidative and proteolytic stress. Redox Biol. 2020; 31:101488. 32201219 10.1016/j.redox.2020.101488 PMC7212483

[22] Muscogiuri G, Barrea L, Somma CD, Laudisio D, Salzano C, Pugliese G, et al. Sex differences of vitamin D status across BMI classes: An observational prospective cohort study. Nutrients. 2019; 11(12):3034. 31842281 10.3390/nu11123034 PMC6950363

[23] Holick MF, Binkley NC, Bischoff-Ferrari HA, Gordon CM, Hanley DA, Heaney RP, et al. Evaluation, treatment, and prevention of vitamin D deficiency: An Endocrine Society clinical practice guideline. J Clin Endocrinol Metab. 2011; 96(7):1911–1930. 21646368 10.1210/jc.2011-0385

[24] Ogan D, Pritchett K. Vitamin D and the athlete: risks, recommendations, and benefits. Nutrients. 2013; 5(6):1856–1868. 23760056 10.3390/nu5061856 PMC3725481

[25] Jung HC, Seo M-W, Lee S, Kim SW, Song JK. Vitamin D_3_ supplementation reduces the symptoms of upper respiratory tract infection during winter training in vitamin D-insufficient taekwondo athletes: a randomized controlled trial. Int J Env Res Pub He. 2018; 15(9):2003. 10.3390/ijerph15092003 PMC6164435 30223447

[26] Lee MC, Ho CS, Hsu YJ, Huang CC. Live and heat-killed probiotic Lactobacillus Paracasei PS23 accelerated the improvement and recovery of strength and damage biomarkers after exercise-induced muscle damage. Nutrients. 2022; 14(21):4563. 36364825 10.3390/nu14214563 PMC9658587

[27] Alessio HM, Hagerman AE, Fulkerson BK, Ambrose J, Rice RE, Wiley RL. Generation of reactive oxygen species after exhaustive aerobic and isometric exercise. Med Sci Sports Exerc. 2000; 32(9):1576–1581. 10994907 10.1097/00005768-200009000-00008

[28] Paschalis V, Nikolaidis MG, Fatouros IG, Giakas G, Koutedakis Y, Karatzaferi C, et al. Uniform and prolonged changes in blood oxidative stress after muscle-damaging exercise. In Vivo. 2007; 21(5):877–883. 18019428

[29] Henning T, Weber D. Redox biomarkers in dietary interventions and nutritional observation studies – From new insights to old problems. Redox Biol. 2021; 41:101922. 33756398 10.1016/j.redox.2021.101922 PMC8020480

[30] Bongiovanni T, Genovesi F, Nemmer M, Carling C, Alberti G, Howatson G. Nutritional interventions for reducing the signs and symptoms of exercise-induced muscle damage and accelerate recovery in athletes: current knowledge, practical application and future perspectives. Eur J Appl Physiol. 2020; 120(9):1965–1996. 32661771 10.1007/s00421-020-04432-3

[31] Huang CC, Lin TJ, Lu YF, Chen CC, Huang CY, Lin WT. Protective effects of L-arginine supplementation against exhaustive exercise-induced oxidative stress in young rat tissues. Chin J Physiol. 2009; 52(5):306–315. 20034235 10.4077/cjp.2009.amh068

[32] Nobari H, Nejad HA, Kargarfard M, Mohseni S, Suzuki K, Carmelo Adsuar J, et al. The effect of acute intense exercise on activity of antioxidant enzymes in smokers and non-smokers. Biomolecules. 2021; 11(2):171. 33513978 10.3390/biom11020171 PMC7910903

[33] Lin AM, Chen K, Chao P. Antioxidative effect of vitamin D_3_ on zinc-induced oxidative stress in CNS. Ann N Y Acad Sci. 2005; 1053(1):319–329. 16179538 10.1196/annals.1344.028

[34] Mokhtari Z, Hekmatdoost A, Nourian M. Antioxidant efficacy of vitamin D. J Parathyr Dis. 2016; 5(1):11–16.

[35] Abdrabbo M, Birch CM, Brandt M, Cicigoi KA, Coffey SJ, Dolan CC, et al. Vitamin D and COVID-19: A review on the role of vitamin D in preventing and reducing the severity of COVID-19 infection. Protein Sci. 2021; 30(11):2206–2220. 34558135 10.1002/pro.4190 PMC8521296

[36] Hoseini R, Rahim HA, Ahmed JK. Concurrent alteration in inflammatory biomarker gene expression and oxidative stress: how aerobic training and vitamin D improve T2DM. BMC Complement Altern Med. 2022; 22(1):1–13. 10.1186/s12906-022-03645-7 PMC9214191 35733163

[37] Heaney RP, Armas LA, Shary JR, Bell NH, Binkley N, Hollis BW. 25-Hydroxylation of vitamin D_3_: Relation to circulating vitamin D_3_ under various input conditions. Am J Clin Nutr. 2008; 87(6):1738–1742. 18541563 10.1093/ajcn/87.6.1738

[38] Todd J, Madigan S, Pourshahidi K, McSorley E, Laird E, Healy M, et al. Vitamin D status and supplementation practices in elite Irish athletes: an update from 2010/2011. Nutrients. 2016; 8(8):485. 27517954 10.3390/nu8080485 PMC4997398

[39] Savolainen L, Timpmann S, Mooses M, Mäestu E, Medijainen L, Tõnutare L, et al. Vitamin D supplementation does not enhance resistance training-induced gains in muscle strength and lean body mass in vitamin D deficient young men. Eur J Appl Physiol. 2021; 121(7):2077–2090. 33821332 10.1007/s00421-021-04674-9

